# Gastric Cancer Peritoneal Carcinomatosis Risk Score

**DOI:** 10.1245/s10434-019-07624-0

**Published:** 2019-07-25

**Authors:** Liang Ji, Matthew J. Selleck, John W. Morgan, Jane Xu, Blake D. Babcock, David Shavlik, Nathan R. Wall, William H. Langridge, Sharon S. Lum, Carlos A. Garberoglio, Mark E. Reeves, Naveenraj Solomon, Jukes P. Namm, Maheswari Senthil

**Affiliations:** 1 School of Public Health, Loma Linda University, Loma Linda, CA USA; 2 Division of Surgical Oncology, Loma Linda University Health, Loma Linda, CA USA; 3 Surveillance, Epidemiology and End Results (SEER) Cancer Registry of Greater California and California Cancer Registry, Sacramento, Loma Linda, CA USA; 4 Division of Biochemistry, School of Medicine, Loma Linda University, Loma Linda, CA USA

## Abstract

**Background:**

Gastric cancer (GC) peritoneal carcinomatosis (PC) is associated with a poor prognosis. Although grade, histology, and stage are associated with PC, the cumulative risk of PC when multiple risk factors are present is unknown. This study aimed to develop a cumulative GCPC risk score based on individual demographic/tumor characteristics.

**Methods:**

Patient-level data (2004–2014) from the California Cancer Registry were reviewed by creating a keyword search algorithm to identify patients with gastric PC. Multivariable logistic regression was used to assess demographic/tumor characteristics associated with PC in a randomly selected testing cohort. Scores were assigned to risk factors based on beta coefficients from the logistic regression result, and these scores were applied to the remainder of the subjects (validation cohort). The summed scores of each risk factor formed the total risk score. These were grouped, showing the percentages of patients with PC.

**Results:**

The study identified 4285 patients with gastric adenocarcinoma (2757 males, 64.3%). The median age of the patients was 67 years (interquartile range [IQR], 20 years). Most of the patients were non-Hispanic white (*n* = 1748, 40.8%), with proximal (*n* = 1675, 39.1%) and poorly differentiated (*n* = 2908, 67.9%) tumors. The characteristics most highly associated with PC were T4 (odds ratio [OR], 3.12; 95% confidence interval [CI], 2.19–4.44), overlapping location (OR 2.27; 95% CI 1.52–3.39), age of 20–40 years (OR 3.42; 95% CI 2.24–5.21), and Hispanic ethnicity (OR 1.86; 95% CI 1.36–2.54). The demographic/tumor characteristics used in the risk score included age, race/ethnicity, T stage, histology, tumor grade, and location. Increasing GCPC score was associated with increasing percentage of patients with PC.

**Conclusion:**

Based on demographic/tumor characteristics in GC, it is possible to distinguish groups with varying odds for PC. Understanding the risk for PC based on the cumulative effect of high-risk features can help clinicians to customize surveillance strategies and can aid in early identification of PC.

Gastric cancer (GC) is the fifth most common malignancy and the third leading cause of cancer death in the world.^1^ The 5-year relative survival is 68.1% for localized, 30.6% for regional, and 5.2% for distant (metastatic) stage gastric cancer.^2^ The peritoneum is a common site of metastasis in gastric cancer, and approximately 15% of the patients have a diagnosis of peritoneal carcinomatosis (PC) at presentation.^3^,^4^ An additional 15–52% go on to experience PC as a result of treatment failure.^3^–^8^ Yang et al.^8^ observed that 52.4% of the treatment-related failures among patients treated with D2 gastrectomyand adjuvant chemoradiation had PC as a single pattern of dissemination.

Whether identified at initial presentation or at progression, PC is associated with a bleak survival of 2.8–4.0 months.^3^,^4^,^9^ In the Evolution of Peritoneal Carcinomatosis (EVOCAPE-1) study,  patients with synchronous and metachronous gastric PC had median survivals of 2.8 months and 3.1 months, respectively.^9^

Despite recent advances in systemic treatment of gastric PC, only marginal survival benefit has been demonstrated. Cytoreductive surgery (CRS) and hyperthermic intraperitoneal chemotherapy (HIPEC) may be an option for select patients. Although CRS ± HIPEC has not been widely accepted due to mixed results, it is well established that patients with a low peritoneal cancer burden who undergo a complete cytoreduction achieve the greatest survival benefit.^8^,^10^–^12^

Prediction of PC risk is critical to identification of patients with limited burden of peritoneal disease. Although grade, histology, and depth of tumor invasion are associated with PC, the cumulative risk for PC when multiple risk factors are present is currently unknown.^3^–^5^ We therefore sought to develop a cumulative PC risk score based on individual demographic and tumor characteristics.

## Methods

**Fig. 1:**
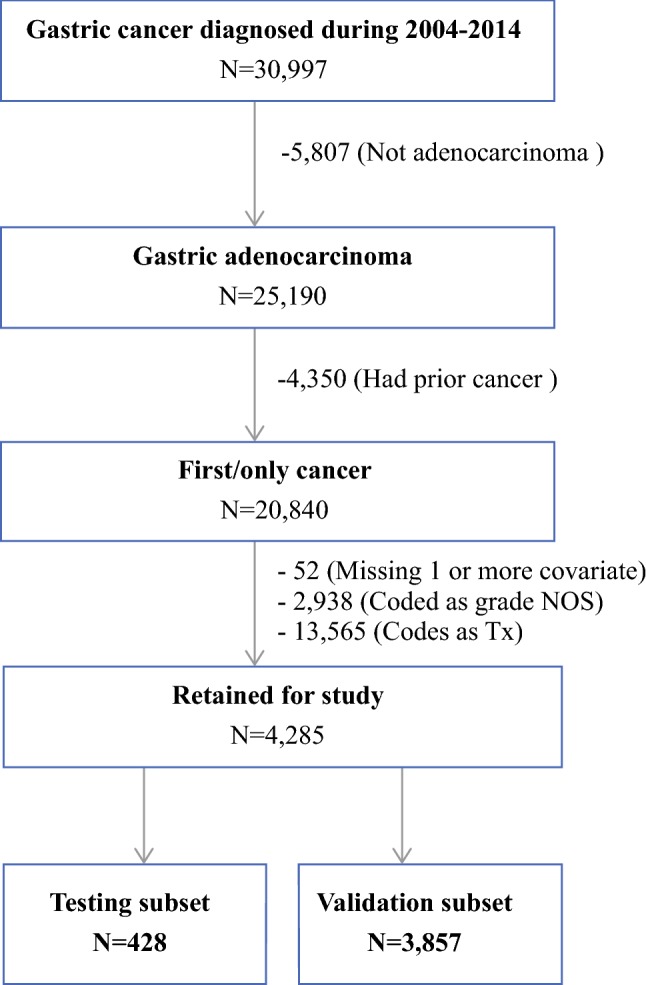
Flowchart of study subject selection

This research involved collaboration between Loma Linda University and California Cancer Registry (CCR) researchers. The CCR is the state-mandated cancer surveillance system that collects, organizes, and analyzes demographic and tumor-specific information for all cancers diagnosed among California residents. The following data were used to identify patients 18 years of age or older with a diagnosis of gastric adenocarcinomas (C16.0–C16.9) between 2004 and 2014: M-8140, M-8143, M-8144, M-8145, M-8210, M-8211, M-8221, M-8255, M-8260, M-8261, M-8262, M-8263, M-8480, M-8481, M-8490.^13^,^14^ Patients with missing data for one or more of the following covariates were excluded from the study: clinical T, tumor grade, anatomic subsite, histology, age, sex, and race/ethnicity. The study subjects were randomly divided into testing (*n* = 428) and validation (*n* = 3857) cohorts. Figure 1 presents the study selection inclusion and exclusion counts.

### Study Variables and Validation Data Set

Eureka is a non-research data management system developed by the CCR to review, consolidate, and accession detailed information for patients receiving cancer diagnosis and care in California, most of which is not found in the CCR database. The dependent variable, PC, is not available in the CCR research database.

To identify patients with PC in the CCR, we developed a three-step Natural Language Processing (NLP) algorithm to Eureka text-field data identifying PC:yes/no.^15^ Step 1 used NLP keyword searches of Eureka text fields for strings that identified positive PC status based on accepted clinical terminology ("Appendix"). Findings for keywords in strings describing PC, including “no evidence of disease,” “NED,” “neg,” “negative,” “questionable,” “rule out,” “r/o,” “without,” “w/o,” and “(–),” were marked as negative for PC. Step 2 included independent review of Eureka records for a random sample of 300 patients with a diagnosis of gastric adenocarcinoma (2004–2014) by two surgeon coauthors (M.S. and B.B.). These findings were used as the “gold standard” for PC status. Step 3 involved comparison of the NLP findings for PC status with the “gold standard”.

The accuracy of data extracted using NLP was confirmed by comparing the data with results obtained by independent review of the Eureka database by the two surgeon coauthors. The findings were then compared with the actual PC status extracted from Eureka for each patient in the testing cohort. The validation cohort was used to generate a regression equation for likelihood of PC associated with each of the purposefully selected demographic and tumor characteristics assessed.

The independent categories of tumor variables extracted from the CCR research database included clinical T (T1, T2, T3, and T4), grade (1, 2, 3 + 4), and anatomic subsite (proximal including cardia [C16.0] and fundus [C16.1]), body [C16.2], distal including antrum [C16.3] and pylorus [C16.4], overlapping including lesser [C16.5] and greater [C16.6] curvature, and overlapping [C16.8]). Histology included intestinal (M-8144), diffuse (M-8145), mucinous (M-8480 and M-8481), signet ring (M-8490), and adenocarcinoma NOS (Not otherwise specified) (M-8140, M-8143, M-8210, M-8211, M-8221, M-8255, M-8260, M-8261, M-8262, and M-8263).^14^ The independent demographic covariates included age categories (18–39, 40–59, and 60+ years), sex (female*/*male), and race/ethnicity (Asian/other, non-Hispanic black, Hispanic, and non-Hispanic white). All independent variables were a priori selected based on existing literature.^3^,^5^,^16^

### Statistical Analysis

Tenfold cross-validation was used to measure prediction accuracy. The study subjects were randomly divided into 10 subsets, with 9 subsets (90%) used for the validation cohort and 1 subset (10%) used for testing.^17^ Multivariable logistic regression was used on the validation cohort to generate a regression equation predicting peritoneal carcinomatosis (Y/N) with all tumor and demographic variables.^18^ This was repeated 10 times, with rotation of the testing subset. Each study subject in the testing subset with a prediction score higher than 50% was categorized as predicted-PC:yes, with the remainder scored as predicted-PC:no.

A 2 × 2 table was used to compare and calculate agreement between predicted-PC (Y/N) and actual PC (Y/N) derived from the NLP text field data. All tests used two-sided interpretations with critical values of 0.05.

Data analyses were performed using, SAS Software, version 9.4 (SAS Institue Inc., Cary, NC, USA) and RStudio 3.4.5 (R Foundation for Statistical Computing, Vienna, Austria).^19^,^20^ In compliance with institutional review board (IRB), CCR, and Eureka, data were extracted and analyzed within the Region 5 office of the CCR using statewide California data.

### Gastric Cancer Peritoneal Carcinomatosis (GCPC) Risk Score

To simplify calculation, beta coefficients obtained from the logistic regression analyses were rounded to the first decimal place. For every 0.1 increase in beta coefficient, the GCPC score for each tumor and demographic variable was assigned an increment of 0.5, starting from zero. Each patient was assigned a total GCPC score as the sum of the GCPC scores for each of the tumor and demographic variables (Table 3). These scores were correlated with odds of PC and grouped into five categories.

## Results

Based on the selection criteria, 4285 gastric adenocarcinoma patients were eligible for the study and further divided into testing and validation cohorts (Fig. 1). Tumor and demographic variables by PC status are presented in Table 1. The majority of the patients were older (> 60 years) and male. Hispanic and Asian/other race/ethnic groups comprised 50% of the study population. The tumor characteristics showed a high proportion of poorly differentiated or undifferentiated cancers.

**Table 1:** Counts (*n*) and column percentages of study subjects with and without peritoneal carcinomatosis (PC) by tumor and demographic variables

	No PC		PC	
	*n*	%	*n*	%
Age (years)				
18–39	136	3.48	49	13.06
40–59	992	25.37	163	43.47
60+	2782	71.15	163	43.47
Sex				
Male	2533	64.78	224	59.73
Female	1377	35.22	151	40.27
Race/ethnicity				
Asian/other	1003	25.65	83	22.13
Non-Hispanic black	212	5.42	24	6.40
Hispanic	1035	26.47	180	48.00
Non-Hispanic white	1660	42.46	88	23.47
Clinical T				
T1	1138	29.11	57	15.20
T2	797	20.38	59	15.73
T3	1322	33.81	100	26.67
T4	653	16.70	159	42.40
Histology type				
Intestinal	542	13.86	28	7.47
Diffuse	227	5.81	41	10.93
Signet ring	720	18.41	138	36.80
Mucinous	70	1.79	7	1.87
NOS	2351	60.13	161	42.93
Anatomic subsite				
Proximal	1597	40.84	78	20.80
Body	1071	27.39	122	32.53
Distal	901	23.05	105	28.00
Overlapping	341	8.72	70	18.67
Grade				
Well-differentiated	221	5.66	5	1.33
Moderately differentiated	1102	28.18	49	13.07
Poorly differentiated or undifferentiated	2587	66.16	321	85.60

*NOS* Not otherwise specified

Findings from NLP review of gastric cancer patients randomly selected from the CCR research database relative to the physician “gold standard” showed a sensitivity of 88% and a specificity of 95%.

The independent PC (yes/no) odds ratios (ORs) for age at diagnosis, sex, race/ethnicity, clinical T stage, histology, anatomic subsite, and tumor grade are presented in Table 2 for the first logistic model. Each of the 10 logistic regression models with purposeful selection identified the same independent variables for model inclusion. The characteristics most highly associated with PC included T4 versus T1 (OR 3.12; 95% CI 2.19–4.44), signetring versus intestinal histology (OR 1.99; 95% CI 1.22–3.24), overlapping versus proximal anatomic subsite (OR 2.27; 95% CI 1.52–3.39), Hispanic versus non-Hispanic white ethnicity (OR 1.86; 95% CI 1.36–2.54), and age of 18–39 years versus 60+ years (OR 3.42; 95% CI 2.24–5.21).

**Table 2:** Adjusted odds ratio (OR) with 95% confidence interval (CI) and *p* value for selected tumor and demographic characteristics as indicators of peritoneal carcinomatosis among gastric cancer patients

	OR	95% CI	*p* value
Age (years)			
18–39 versus 60+	3.42	2.24–5.21	< 0.001
40–59 versus 60+	1.90	1.46–2.46	< 0.001
Sex			
Female versus male	0.96	0.75–1.23	0.737
Race/ethnicity			
Asian/other versus non-Hispanic white	1.16	0.81–1.65	0.424
Non-Hispanic black versus non-Hispanic white	1.61	0.95–2.71	0.075
Hispanic versus non-Hispanic white	1.86	1.36–2.54	< 0.001
Clinical T			
T2 versus T1	1.19	0.79–1.79	0.403
T3 versus T1	1.28	0.89–1.85	0.182
T4 versus T1	3.12	2.19–4.44	< 0.001
Histology			
Diffuse versus intestinal	1.70	0.96–3.02	0.070
Mucinous versus intestinal	1.42	0.54–3.73	0.481
NOS versus intestinal	1.22	0.78–1.93	0.384
Signet ring versus Intestinal	1.99	1.22–3.24	0.006
Anatomic subsite			
Body versus proximal	1.64	1.15–2.34	0.006
Distal versus proximal	1.63	1.16–2.30	0.005
Overlapping versus proximal	2.27	1.52–3.39	< 0.001
Grade			
Moderately versus well-differentiated	1.42	0.55–3.70	0.467
Poorly differentiated or undifferentiated versus well-differentiated	2.22	0.88–5.59	0.092

*NOS* Not otherwise specified

The risk factor scores for demographic/tumor characteristics were correlated with odds of PC and grouped into five categories. The incremental increase in risk score was associated with increasing odds of PC. The percentages with PC by total risk score are presented in Table 3.

**Table 3:** Gastric cancer peritoneal carcinomatosis (GCPC) score

Patient and tumor characteristics	Score
Age (years)	
18–39 versus 60+	6
40–59 versus 60+	3
60+	0
Race/ethnicity	
Non-Hispanic white	0
Asian/other	0.5
Non-Hispanic black	3
Hispanic	2.5
Clinical T stage	
T1	0
T2	1
T3	1
T4	5.5
Histology	
Intestinal	0
NOS	1
Mucinous	1.5
Diffuse	2.5
Signet ring	3.5
Tumor location	
Proximal	0
Body	2.5
Distal	2.5
Overlapping	4
Tumor grade	
Well-differentiated	0
Moderately well-differentiated	2
Poorly differentiated or undifferentiated	4

Score	% With PC
0–10	3.2
10.5–14	9.5
14.5–17	15
17.5–20	26.1
20–26	46.4

*NOS* Not otherwise specified

**Fig. 2:**
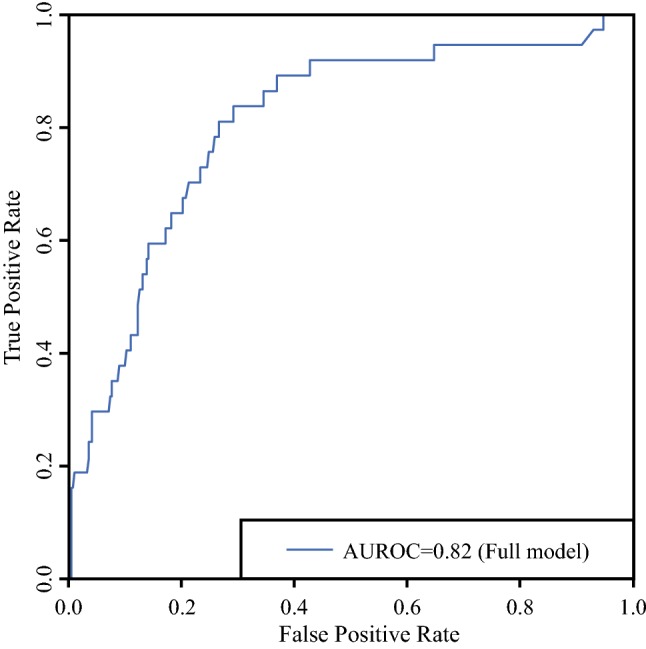
Area under the receiver operating characteristic (AUROC) curve demonstrating performance of the model in comparison to the model without T stage and without T stage and subsite

Findings from the comparison of predicted versus actual PC status showed 92.2% agreement with the area under the receiver operator characteristics (ROC) curve of 0.82 (Fig. 2).

## Discussion

We have created a cumulative GCPC risk score based on tumor and demographic variables available at the time of diagnosis that could be incorporated into clinical practice to guide surveillance and management strategies in GC. This score is being developed further into a publicly available nomogram.

Our model used NLP to glean relevant detailed information not available as discrete variables within the CCR research database. In addition to creation of a risk score, our results also highlight racial/ethnic differences in risk for PC, depicted by 86% higher odds for PC in Hispanics than in non-Hispanic white GC patients.

To the best of our knowledge, this is the first report to demonstrate that Hispanic ethnicity is an independent predictor of PC in GC. Factors identified as associated with PC in this study were congruent with findings reported in the literature.^5^,^16^ D’Angelica et al.^5^ reported on 11,172 patients who underwent an R0 resection from 1985 to 2000. Of these patients, 29% had peritoneal recurrence. Advanced T stage, distal location, diffuse subtype, and female sex each were predictive of PC.

Thomassen et al.^3^ found that between 1995 and 2011 in the Netherlands, metastatic disease was present in 39% of patients at presentation. The findings showed PC present in 14% of all GC patients and metastatic disease in 35% of these patients. Younger age (< 60 years), female gender, advanced T and N stage, signet ring or linitis plastica, and primary tumors of overlapping locations all were associated with higher odds for PC development.

In a study of 550 patients with GC who underwent definitive resection, Seyfried et al.^4^ identified grade 3/4 (OR 2.03; 95% CI 3.65–1.13), nodal positivity (OR 2.39; 95% CI 4.26–1.34), signet-ring cell (OR 3.88; 95% CI 9.71–1.56), and T3/4 (OR 2.35; 95% CI 1.35–4.12) to be independent risk factors for the development of metachronous PC. Although these factors have been recognized as predictors of PC, the cumulative risk score presented in this study, using multiple clinical and demographic variables, provides valuable information for tailoring surveillance strategies for those at highest risk for PC.

In contrast to the results reported by Thomassen et al.^3^ and D’Angelica et al.^5^ female sex was not an independent predictor of PC in the current study. Our findings showed that the slightly higher odds for GCPC among females was diminished to a near null finding when the anatomic subsite was adjusted. Additional stratification by anatomic subsite and sex (not presented in the tables) showed that only 25.1% of the female patients had proximal GC versus 46.9% of the males, which in our study was less likely to be associated with PC. These findings underscore the need for further investigation of the reason for the anatomic subsite difference in GC observed between the sexes.

Hispanics, compared to non-Hispanic whites, had a nearly twofold increase in the odds for PC (OR 1.86; 95% CI 1.36–2.54; *p* < 0.001) after adjustment for other covariates (Table 2). Recent studies have shown an increase in annual incidence of GC in Hispanics, particularly among young men.^21^,^22^ This concerning trend currently is compounded by our observation that Hispanic ethnicity is an independent risk factor for PC. To our knowledge, our study is the first to show this association. Inclusion of race/ethnic differences, which have been central to GC discussions for decades, should be reflected in surveillance strategies. Although our overall study population consisted of nearly 30% Hispanics, this certainly differs from the overall demographics for the remainder of the country, with reported incidence rates of 10–18%.^21^,^23^

Previous population-based studies from both California and Texas have noted an increased prevalence of *Helicobacter pylori* infection and risk of gastric cancer for this group.^24^,^25^ Therefore, the applicability of ethnicity as a risk factor in our study needs further validation in the general U.S. population. Nevertheless, Hispanic ethnicity was a strong predictor of PC even when controlled for other tumor factors. Environmental, social, and access issues could have been contributing to this observation. Additionally, Hispanic and non-Hispanic gastric cancers may have genomic differences, all of which warrant further work.

Various surveillance strategies and their impact on survival have been investigated previously.^26^–^29^ In 2014, an international roundtable of 32 experts from 12 countries reached a consensus that currently available data do not demonstrate a survival improvement with intensive surveillance.^29^ However, most surveillance strategies are based on the assumption that patients with GC are sufficiently staged by tumor-node-metastasis (TNM) variables.

A key observation of our study was the ability to identify increased risk of PC in an individual patient. The majority of the risk factors incorporated into the GCPC score (clinical T stage, grade, anatomic subsite, and presence or absence of signet-ring histology) should be readily available to clinicians at the time of initial diagnosis and may help tailor management. Although short-interval imaging or diagnostic laparoscopy might be useful, the percentage of increased risk that warrants change in surveillance strategies needs prospective clinical study. In addition, advances in our understanding of the molecular subtypes of gastric cancer likely will allow further stratification based on risk. In future studies, molecular information could be added to the known risk factors to improve the predictive power of the model.

Early detection of recurrence allows intervention at a time when treatment options currently available have potential to improve survival. As coming years bring advancements in therapeutic options, a tailored surveillance strategy based on PC risk could result in meaningful improvement in patient survival.

## Study Limitations

This study was subject to the biases inherent in database research such as selection, reporting, and time-dependent biases. However, we do not believe these would change the direction of our findings.

Among the 20,840 gastric adenocarcinoma patients in California (2004–2014), 13,565 were coded as Tx, 2938 were classified as unspecified grade, and 52 were missing one or more demographic or other tumor characteristics. Classification as Tx is consistent with the rapid progression of gastric cancer to advanced T stage and the limited value of T-stage information at the time of late presentation.

Generalization of the findings presented in this report should be limited to gastric PC patients with complete demographic and tumor characteristics. Nevertheless, it is reasonable to assume that the majority of patients classified as Tx actually were T4. Based on this assumption, it seems reasonable to extend the findings presented in Table 2 to Tx patients.

Although nodal status is a strong predictor of PC, clinical nodal status was not used in our PC risk score model due to wide inter-observer variability and inconsistent reporting of clinical nodal status in CCR. As with T stage, the perceived limited value of N stage information at the time the patient presents with metastases may have obviated recording by treating physicians. However, due to the prognostic value of clinical nodal status, even in PC, it is important to assign and report it accurately.^30^ A future validation study using a prospective data set would allow us to define the weight of nodal status in the GCPC risk score.

In addition, due to the retrospective nature and timing of the study, more granular information such as human epidermal growth factor receptor 2 (HER2)-Neu status and cytology-positive M1 disease was not available. Also, CCR does not allow distinction between synchronous and metachronous PC. However, for the purpose of this study, we focused on the presence of PC.

Our model of association was tested against a subgroup that was naïve to the regression findings. Validity might be different if the model is tested with an additional data set. However, our data set represents one of the largest and most diverse cohorts of GC in the United States, enhancing the generalizability of the reported findings.

## Conclusions

This GCPC risk score uses readily available tumor and demographic variables to create a cumulative risk score for PC, which in turn can be used by clinicians to customize surveillance strategies.

## Appendix

Natural language processing keyword searches of Eureka text fields for the strings that identified positive peritoneal carcinomatosis status included the following: diaphragm carcinomatosis, diaphragm implants, diaphragm mass, diaphragm metastasis, diaphragm metastases, diaphragm mets, diaphragm studding, mesenteric caking, mesenteric carcinomatosis, mesenteric infiltration, mesenteric implants, mesenteric mass, mesenteric metastasis, mesenteric metastases, mesenteric mets, mesenteric studding, omental caking, omental carcinomatosis, omental infiltration, omental implants, omental mass, omental metastasis, omental metastases, omental mets, omental studding, peritoneal caking, peritoneal carcinomatosis, peritoneal implants, peritoneal mass, peritoneal metastasis, peritoneal metastases, peritoneal mets, peritoneal studding, and seeding.

## References

[1] Bray F, Ferlay J, Soerjomataram I, Siegel RL, Torre LA, Jemal A (2018). Global cancer statistics 2018: GLOBOCAN estimates of incidence and mortality worldwide for 36 cancers in 185 countries. CA Cancer J Clin..

[2] Noone AM HN, Krapcho M, Miller D, Brest A, Yu M, Ruhl J, et al (eds). SEER Cancer Statistics Review, 1975–2015, based on November 2017 SEER data submission, posted to the SEER web site April; 2018. https://seer.cancer.gov/csr/1975_2015/. Accessed 12 Jan 2019.

[3] Thomassen I, van Gestel YR, van Ramshorst B (2014). Peritoneal carcinomatosis of gastric origin: a population-based study on incidence, survival, and risk factors. Int J Cancer..

[4] Seyfried F, von Rahden BH, Miras AD (2015). Incidence, time course, and independent risk factors for metachronous peritoneal carcinomatosis of gastric origin: a longitudinal experience from a prospectively collected database of 1108 patients. BMC Cancer..

[5] D’Angelica M, Gonen M, Brennan MF, Turnbull AD, Bains M, Karpeh MS (2004). Patterns of initial recurrence in completely resected gastric adenocarcinoma. Ann Surg..

[6] Roviello F, Marrelli D, de Manzoni G (2003). Prospective study of peritoneal recurrence after curative surgery for gastric cancer. Br J Surg..

[7] Spolverato G, Ejaz A, Kim Y (2014). Rates and patterns of recurrence after curative intent resection for gastric cancer: a United States multi-institutional analysis. J Am Coll Surg..

[8] Yang XJ, Li Y, Yonemura Y (2010). Cytoreductive surgery plus hyperthermic intraperitoneal chemotherapy to treat gastric cancer with ascites and/or peritoneal carcinomatosis: results from a Chinese center. J Surg Oncol..

[9] Sadeghi B, Arvieux C, Glehen O (2000). Peritoneal carcinomatosis from non-gynecologic malignancies: results of the EVOCAPE 1 multicentric prospective study. Cancer..

[10] Sayag-Beaujard AC, Francois Y, Glehen O (1999). Intraperitoneal chemo-hyperthermia with mitomycin C for gastric cancer patients with peritoneal carcinomatosis. Anticancer Res..

[11] Yonemura Y, Canbay E, Endou Y (2014). Peritoneal cancer treatment. Expert Opin Pharmacother..

[12] Sugarbaker PH, Yu W, Yonemura Y (2003). Gastrectomy, peritonectomy, and perioperative intraperitoneal chemotherapy: the evolution of treatment strategies for advanced gastric cancer. Semin Surg Oncol..

[13] California Cancer Registry. Retrieved 23 January 2019 at https://www.ccrcal.org/learn-about-ccr/.

[14] Fritz APC, Jack A, Shanmugaratnam K, Sobin L, Parkin DM, Whelan S (2000). International Classification of Diseases for Oncology.

[15] SAS Natural Language Processing. Retrieved 23 January 2919 at https://www.sas.com/en_us/insights/analytics/what-is-natural-language-processing-nlp.html.

[16] Ohi M, Mori K, Toiyama Y (2015). Preoperative prediction of peritoneal metastasis in gastric cancer as an indicator for neoadjuvant treatment. Anticancer Res..

[17] Kuhn M, Johnson K (2013). Applied Predictive Modeling.

[18] Bursac Z, Gauss CH, Williams DK, Hosmer DW (2008). Purposeful selection of variables in logistic regression. Source Code Biol Med..

[19] Inc SI. *SAS/STAT 14.1 User’s Guide.* SAS Institute Inc., Cary, NC, 2015.

[20] RCoreTeam. R: A language and environment for statistical computing. R Foundation for Statistical Computing, 2018. https://www.R-project.org/. Accessed 1 Oct 2018.

[21] Merchant SJ, Kim J, Choi AH, Sun V, Chao J, Nelson R (2017). A rising trend in the incidence of advanced gastric cancer in young Hispanic men. Gastric Cancer..

[22] Chang ET, Gomez SL, Fish K (2012). Gastric cancer incidence among Hispanics in California: patterns by time, nativity, and neighborhood characteristics. Cancer Epidemiol Biomarkers Prev..

[23] Gupta S, Tao L, Murphy JD (2019). Race/ethnicity-, socioeconomic status-, and anatomic subsite-specific risks for gastric cancer. Gastroenterology..

[24] Rajabi B, Corral JC, Hakim N, Mulla ZD (2012). Descriptive epidemiology of gastric adenocarcinoma in the state of Texas by ethnicity: Hispanic versus white non-Hispanic. Gastric Cancer..

[25] Dong Elizabeth, Duan Lewei, Wu Bechien U. (2017). Racial and Ethnic Minorities at Increased Risk for Gastric Cancer in a Regional US Population Study. Clinical Gastroenterology and Hepatology.

[26] Bohner H, Zimmer T, Hopfenmuller W, Berger G, Buhr HJ (2000). Detection and prognosis of recurrent gastric cancer: is routine follow-up after gastrectomy worthwhile?. Hepatogastroenterology..

[27] Kodera Y, Ito S, Yamamura Y (2003). Follow-up surveillance for recurrence after curative gastric cancer surgery lacks survival benefit. Ann Surg Oncol..

[28] Eom BW, Ryu KW, Lee JH (2011). Oncologic effectiveness of regular follow-up to detect recurrence after curative resection of gastric cancer. Ann Surg Oncol..

[29] Laks S, Meyers MO, Kim HJ (2017). Surveillance for gastric cancer. Surg Clin North Am..

[30] Zhou R, Wu Z, Zhang J (2016). Clinical significance of accurate identification of lymph node status in distant metastatic gastric cancer. Oncotarget..

